# Volunteer motivators for participating in HIV vaccine clinical trials in Nairobi, Kenya

**DOI:** 10.1371/journal.pone.0183788

**Published:** 2017-09-07

**Authors:** Borna A. Nyaoke, Gaudensia N. Mutua, Rose Sajabi, Delvin Nyasani, Marianne W. Mureithi, Omu A. Anzala

**Affiliations:** KAVI -Institute of Clinical Research (KAVI-ICR), University of Nairobi. Nairobi, Kenya; Cincinnati Children's Hospital Medical Center, UNITED STATES

## Abstract

**Background:**

1.5 million Kenyans are living with HIV/AIDS as per 2015 estimates. Though there is a notable decline in new HIV infections, continued effort is still needed to develop an efficacious, accessible and affordable HIV vaccine. HIV vaccine clinical trials bear risks, hence a need to understand volunteer motivators for enrolment, retention and follow-up. Understanding the factors that motivate volunteers to participate in a clinical trial can help to strategize, refine targeting and thus increase enrolment of volunteers in future HIV vaccine clinical trials. The health belief model classifies motivators into social benefits such as ‘advancing research’ and collaboration with science, and personal benefits such as health benefits and financial interests.

**Method:**

A thematic analysis was carried out on data obtained from four HIV clinical trials conducted at KAVI-Institute of Clinical Research in Nairobi Kenya from 2009 to 2015. Responses were obtained from a Questionnaire administered to the volunteers during their screening visit at the research site.

**Results:**

Of the 281 healthy, HIV-uninfected volunteers participating in this study; 38% were motivated by personal benefits including, 31% motivated by health benefits and 7% motivated by possible financial gains. In addition, 62% of the volunteers were motivated by social benefits with 20% of who were seeking to help their family/society/world while 42% were interested in advancing research.

**Conclusion:**

The majority of volunteers in the HIV vaccine trials at our site were motivated by social benefits, suggesting that altruism can be a major contributor to participation in HIV vaccine studies. Personal benefits were a secondary motivator for the volunteers. The motivators to volunteer in HIV clinical trials were similar across ages, education level and gender. Education on what is needed (including volunteer participation) to develop an efficacious vaccine could be the key to greater volunteer motivation to participate in HIV vaccine clinical trials.

## Introduction

Kenya is ranked jointly with Mozambique and Uganda as having the fourth largest HIV epidemic in the world. There are over 1.5 million Kenyans living with HIV/AIDS as of 2015 estimates, with almost 660,000 children orphaned due to AIDS [1]. There has been a notable decline in HIV prevalence in Kenya due to increased access to Anti-Retroviral Treatment (ART) and significant changes in sexual behaviour. The incidence of new infections has also declined by almost 16%, from 116,349 in 2009 to 100,501 in 2013 [2]. The number of people dying from AIDS-related causes has also reduced by more than 50% from 2005 to 2011 [3]. Women still face a higher risk of HIV infection and a shorter life expectancy than men. With a 6.9% prevalent rate they make up a higher proportion of those living with HIV as compared to 4.4% in men [4]. Kenya aims at reducing new HIV infections by 75% and AIDS related mortality by 25% between 2015 and 2019 [5]. An efficacious, affordable and accessible HIV vaccine would go a long way in achieving these goals.

Clinical trials involve the active participation of volunteers to capture data, which may interfere with their time management and daily schedules, as vaccine trials may take years to study the various long-term immunological responses crucial in determining the vaccines efficacy and efficiency. However, the use of human subjects in these studies as a unit of analysis introduces certain challenges [6]. Studies have been performed in developing countries that have been shown to be less exacting than they would be in the case of research carried out in the sponsoring country which have sometimes unnecessarily exposed subjects to risk [7].

Regulations to protect the human rights of the clinical trial subjects have been elaborated to ensure their integrity and dignity are upheld during their participation in biomedical research. Unfortunately a strong impression has been left by a history of conducting unethical studies in developing countries which are still used as a point of reference. Other factors such as stigma associated with HIV/AIDS also contribute to the challenge of HIV vaccine trial participation [8]. In Kenya, a country still defined by high levels of illiteracy, poverty and poor access to healthcare, it may seem that conducting clinical trials in such a place may create inherent bias. This may be occasioned by systematic selection errors as participation may be limited by understanding of the clinical trial process or interest in the financial or medical incentives offered during clinical trials. Special protection may thus be suggested in developing countries such as Kenya to address these factors [9].

The Kenya National Council for Science Technology and Innovation (NACOSTI), a government authority, usually approves protocols covering clinical research and accredits ethics committees that can approve study protocols. The Kenyatta National Hospital-University of Nairobi (KNH-UoN) ethics and research committee also attracts many research proposals owing to its facilities, and to the diversity of patients that are treated at Kenyatta National Hospital, the largest referral hospital in Kenya. The hospital's ethics and research committee was formed to take charge of all research conducted within the hospital or the College of Health Sciences at the University of Nairobi. The Kenya Medical Research Institute (KEMRI) vets all proposals for research that involve humans. The committee is multi-sectoral and multidisciplinary, with most of its members coming from outside the institute, to ensure its independence. Fortunately, it is clear that there has been increased emphasis on bioethics in Kenya in relation to clinical studies of human subjects which ensures the ethical evaluation process that a clinical study must pass is sufficiently rigorous and is in accordance with national laws [10].

Individuals have little or no sense of ownership over clinical trials as they may have a false belief that the science is beyond their scope of understanding and the practicalities of vaccine development does not necessarily involve them [11]. To enrol such a group of people into a clinical trial, especially a HIV vaccine clinical trial which is a first in humans, there is need to elucidate the motivators that may be required for volunteer enrolment, retention and follow-up in a clinical study [12]. Taken together, the aim of this study is to identify the factors that affect volunteer enrolment into HIV vaccine clinical trials to allow refining and targeting of volunteers by research sites conducting HIV vaccine trials.

This study was nested within three actual Phase I HIV preventive vaccine clinical trials and one Vaccine Preparedness Study (VPS) conducted between 2009 and 2015 at KAVI-Institute of Clinical Research (KAVI-ICR), University of Nairobi, in Nairobi, Kenya. The actual HIV vaccine clinical trials included: B003 study investigating the candidates Ad26.EnvA-01 and Ad35-ENV; B004 was investigating HIV-MAG +GENEVAX® IL-12, Ad35-grin/ENV; and HIVCORE investigating Ad35-GRIN, MVA.HIV-consv, and pSG2.HIVconsv-DNA. K001 was a VPS which assessed the willingness of volunteers to take part in future HIV vaccine clinical trials, while also looking at the chances of these volunteers acquiring HIV under conditions similar to the conditions that would exist in HIV vaccine studies.

## Materials and methods

### Study site

This study was conducted at KAVI-ICR, which has clinics at Kenyatta National Hospital and Kangemi with Nairobi as its catchment population. Nairobi is the capital city of Kenya with a population of 6.54 million people and a HIV prevalence rate of 6.8% which is almost the same as the National rate of 6% [13]. It has approximately 2.5 million slum dwellers in about 200 settlements representing almost 60% of Nairobi’s population [14].

### Study design

A cross-sectional, descriptive, mixed study design with both quantitative and qualitative approaches was used to elicit the volunteer’s motivators for participation in the HIV vaccine clinical trials and the HIV VPS.

### Data collection

The data used in this study was from interviewer-administered questionnaires given to participants of the HIV vaccine clinical trials and the VPS. The questionnaire was semi-structured, containing both open-ended and closed-ended questions. Closed-ended questions were used to obtain demographic data while open-ended questions were used to collect information on their motivation to participate in the clinical trials. This data was collected during their screening visits at the research sites.

### Recruitment of the participants

A convenience sample was used to capture data on 281 volunteers from the KAVI-ICR sites participating in the four clinical trials between the years 2009 to 2015. Those participating in the clinical trials were approached and invited to take part in the interview. Enrolment into this study took place until the last volunteer was screened into the clinical trials in 2015.

Out of the 304 volunteers who took part in these clinical trials over this period of time, only 23 declined to take part in this study; the volunteers were not probed on their reasons for declining. All the volunteers in the four clinical trials were healthy, heterosexual, low risk adults, 18 to 50 years of age, both ages inclusive, participating in HIV vaccine clinical trials or in the VPS at KAVI-ICR from the period of 2009–2015. The volunteers participating in the clinical trials were not provided with any other payment except for reimbursement of costs incurred.

The study was not intended to be representative of the entire population of Kenya but rather designed to identify the main motivators that lead to clinical study participation and whether these motivators differ by variables.

### Ethics statement

The volunteers provided written informed consent to take part in the clinical trials and have information pertaining to their participation in the HIV clinical trials collected over this period. All the study protocols and informed consent documents were approved by the Kenyatta National Hospital- University of Nairobi (KNH-UoN) Ethics Review Board with the following ethics approval numbers: P11/01/2013 (HIVCORE), P277/10/2008 (K001), P167/5/2010 (B003) and P298/07/2011 (B004).

### Data analysis

#### Qualitative analysis

**Fig 1:**
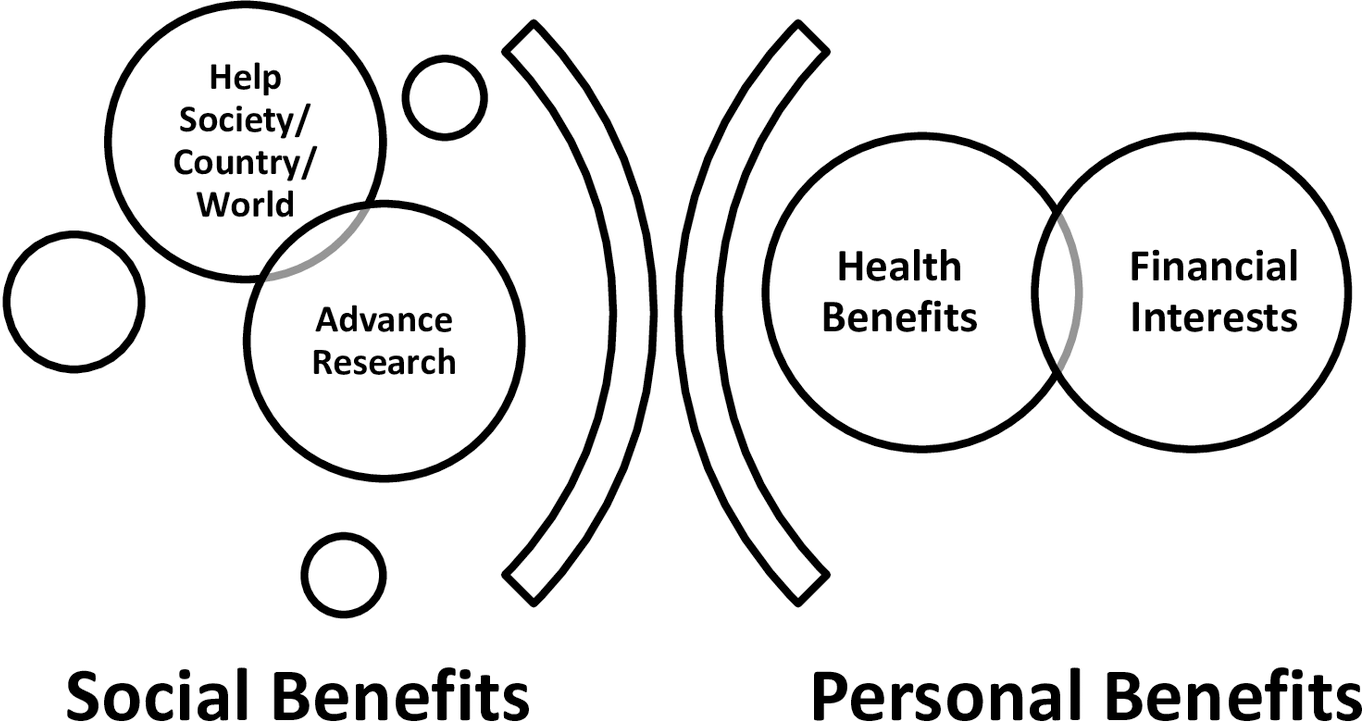
Motivation codes.

Qualitative data was analysed using thematic analysis where responses were grouped into themes. BAN read the responses provided, developed labels representing recurring themes (‘codes’). As key themes began to emerge they were grouped and merged. Subsequent analysis involved joint reading by BAN and the nurse counsellors (RS and DN) followed by long table method to further link, expand and refine themes, until the final results were created; this was done to improve inter-rater reliability [15]. For example, ‘help society/country/world’, and ‘advancing research’ were grouped under ‘social benefits and ‘health benefits’ and ‘financial interests’ were grouped under ‘personal benefits’. The themes developed were then coded and linked up with the quantitative data for further analysis using quantitative techniques. Inductive analysis was applied using Ryan and Bernard strategies [16] as shown in Fig 1.

#### Quantitative analysis

Codes developed from the qualitative data on motivators and quantitative data on demographic characteristics were entered in to SPSS version 21 for analysis. Descriptive statistics such as frequencies, percentages, mean and standard statistics was used to describe occurrences of motivators across various volunteer demographics. Chi-square test and Kruskal Wallis test were used to test the association between volunteer demographics and motivators at 95% confidence interval. Results were presented in form of text, tables and figures.

## Results

### Socio-demographic characteristics

Majority of the volunteers were male while 38.4% were female. Over half of the volunteers had attained secondary education and 21.4% had attained tertiary education. This demonstrates low participation among people with higher levels of education. Most (40.6%) of the volunteers were aged between 18 and 23 years. The average age of the volunteers was 24.8(±0.6) years and as shown in Table 1 below the ages of volunteers was positively skewed, showing that the volunteers were relatively young.

**Table 1:** Socio-demographic characteristics of volunteers.

		Frequency (n)	Percent (%)
Gender of respondent	Male	173	62
	Female	108	38
Education level of respondent	Primary	59	21
	Secondary	162	58
	Tertiary	60	21
Age of respondent in years	< = 23	114	41
	24–28	101	36
	29–33	38	13
	34–38	22	8
	> 38	6	2

### Motivators

Social benefits were the major motivator (62%) to the volunteers. According to majority (42%) of the respondents the desire to contribute to advancement of research which leads to improvement of societal well-being was the primary motivator as shown in Table 2 below.

**Table 2:** Volunteer motivators for participating in HIV vaccine clinical trials.

Motivators	Categories	Frequency (n)	Percent (%)
Personal benefits	Financial interest	19	7
	Health benefits	87	31
	**Total**	**106**	**38**
Social benefits	Advancing research	119	42
	Help society	56	20
	**Total**	**175**	**62**

This revealed that altruism was the most common motive among the clinical trial volunteers as shown by the following excerpts.

“I want to be proud later in life for participating in getting a HIV vaccine if it is successful”. (Male, 21, Single, Secondary education)

“I believe I can help you people [researchers] find a good vaccine so people can stop ‘catching’ and dying from HIV”. (Male, 20, Single, Primary education)

As a social benefit, there was also a motive to participate in HIV vaccine clinical trials in attempts to make a social contribution to those infected by HIV (20%). This was specifically among those with a close person who has been infected by HIV as illustrated by the following excerpts:

“I lost my sister and brother because of HIV and I don’t want to lose anyone else close to me to that horrible disease”. (Female, 22, Married, Secondary education)

“I feel sad when I see people suffering from HIV/AIDS and I think I can help by participating in this study”. (Male, 26, Single, Secondary education)

Personal benefits were also cited by few respondents (38%) although as a secondary motivation. This included the desire to get health benefits (31%) including desire to know their HIV status as illustrated by the following excerpts.

“I would like to know if I am in good health”. (Female, 19, Single, Primary education)

“I heard I will have a full medical examination done by a doctor for free”. (Male, 34, Married, Tertiary education)

As a personal benefit, there was also the desire to get financial benefits although it was the least (6.8%) cited reason for volunteering in the trials as showed by the excerpts below.

“My friend told me she was in another study like this one and she was given money called ‘reimbursement’.” (Female, 25, Single, Primary education)

“I am not working at the moment and I am a single mother of two children so I wouldn’t mind the money that we are going to receive.” (Female, 36, Widowed, Primary education)

**Fig 2:**
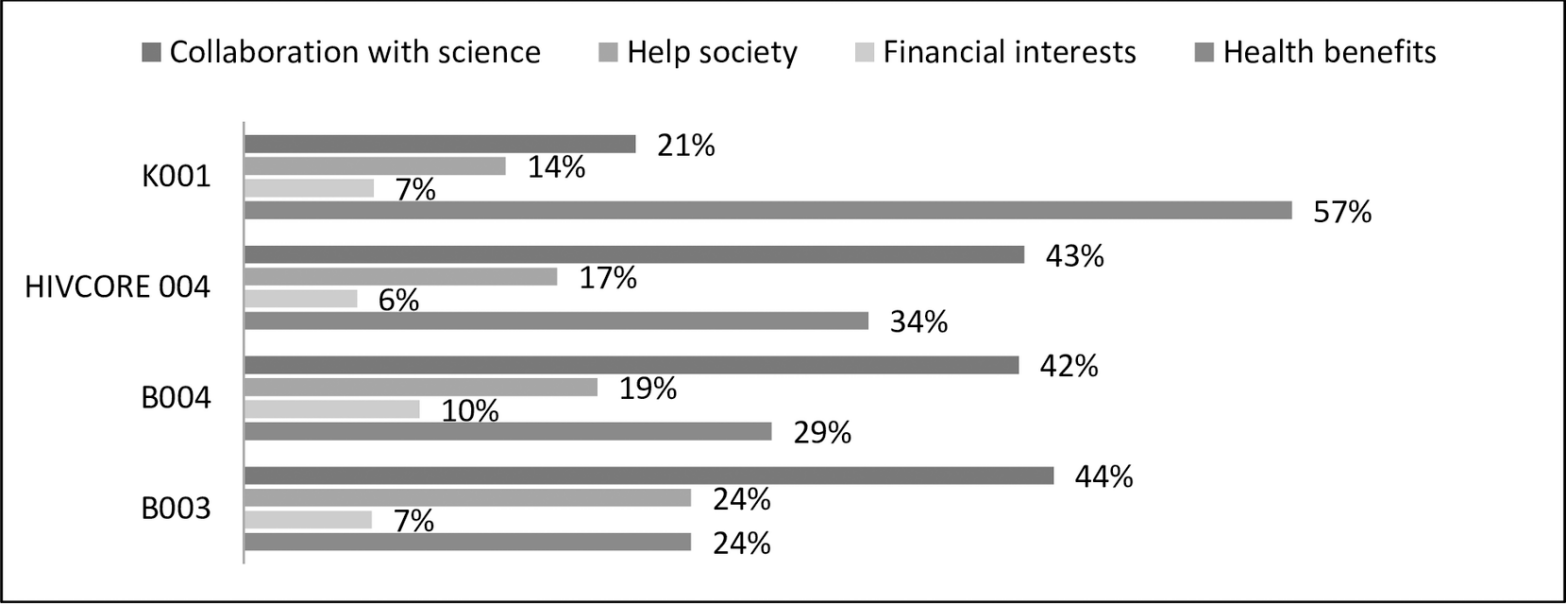
Motivators by trial arms.

Majority of the volunteers in the VPS were motivated by personal benefits (57% health benefits and 7% financial interests) while on the other hand volunteers in the three Phase I trials were mostly consistently motivated by social benefits as indicated in Fig 2. However, Chi-square test of association (χ^2^ = 5.963, df = 3, p = 0.113) indicated that there was no significant variation in motivators among the clinical trial arms. This showed that there was no difference in motivators between Phase I trials and the VPS trial.

#### Motivators by socio-demographic characteristics

**Fig 3:**
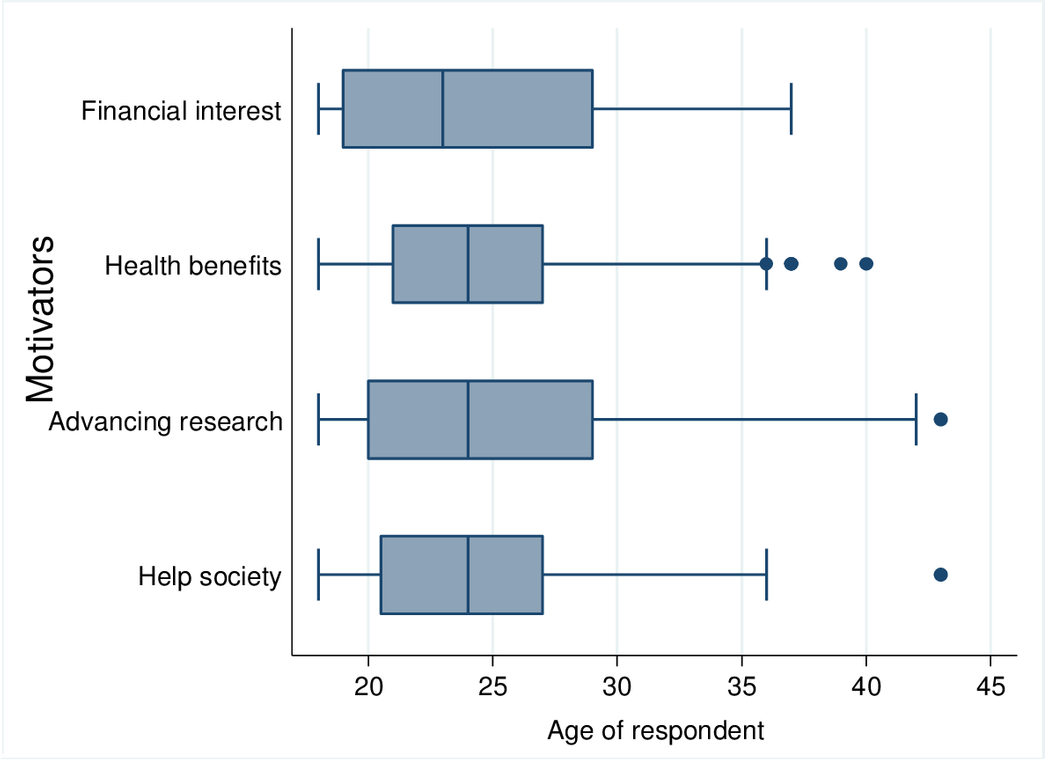
Age and motivators.

Age and motivators. Despite the fact that financial interests motivated younger volunteers, while the need to help society and advance research motivated older volunteers, there were no significant age differences among different motivators (Kruskal Wallis test: χ^2^ = 0.113, df = 3, p = 0.990). The younger volunteers joining the trials due to financial reasons could be attributed to the fact that younger people might not be as financially established and may be looking for ways to earn money. Fig 3 showed that motivators were similar across the different ages of volunteers.

Motivators by gender. As shown in Table 3 below, most male volunteers (45.1%) and female volunteers (38%) were motivated by desire to contribute to advancing research, however there was no significant association between motivators and gender of volunteers (Chi-square test: χ^2^ = 1.598, df = 3, p = 0.660). This indicated that both male and female volunteers were motivated by similar motivators.

**Table 3:** Volunteer motivators by gender.

Motivators	Gender of respondent			
	Male		Female	
	Frequency	Percent (n = 173)	Frequency	Percent (n = 108)
Financial interest	12	7%	7	7%
Health benefits	50	29%	37	34%
Advancing research	78	45%	41	38%
Help society	33	19%	23	21%

Motivators by education level. The desire to advance research was the most common motivator across all education levels (Table 4). Chi-square test of association (**χ**^2^ = 7.430, df = 6, p = 0.283) indicated that there was no significant association between motivators and volunteer education level.

**Table 4:** Volunteer motivators by education level.

Motivators	Education level of respondent					
	Primary		Secondary		Tertiary	
	Frequency	Percent (n = 59)	Frequency	Percent (n = 162)	Frequency	Percent (n = 60)
Financial interest	7	12%	10	6%	2	3%
Health benefits	17	29%	52	32%	18	30%
Advancing research	20	34%	68	42%	31	52%
Help society	15	25%	32	20%	9	15%

## Discussion

An individual must usually believe in both their susceptibility to and severity of a disease for them to effectively change health behaviors, as they provide a clear and present danger. The Health Belief Model proposes that people will respond best to messages about health promotion or disease prevention when the following four conditions for change exist [17]:

1. An individual’s belief of risk of developing a specific condition.
2. Belief that the risk is serious and the consequences of developing the condition are undesirable.
3. Belief that the risk will be reduced by a specific behavioural change.
4. Belief that barriers to the behaviour change can be overcome and managed.

The first condition in the Health Belief Model is perceived threat. If the person does not see behaviour as risky or threatening, there is no stimulus to act. HIV/AIDS is quite prevalent in Kenya and there are high chances of an individual identifying with someone with the disease. This (as Table 5 illustrates) may be a component in participating in a trial that seeks to prevent HIV infection.

**Table 5:** Health belief model components.

Concept	HIV Infection	Participating in HIV vaccine clinical trials
Perceived susceptibility	High risk of acquiring HIV in Nairobi, Kenya.	High risk of exposure to HIV infection in Nairobi, Kenya.
Perceived severity	Consequences of HIV infections require actions to prevent it	HIV vaccine may provide an answer to prevent HIV infections
Perceived benefits	Need to use all methods possible to avoid acquiring HIV	If vaccine works volunteers could be protected from HIV. Financial incentives. Medical care.
Cues to action	Education on abstinence, being faithful and use of condoms	Incentives to participate in clinical trials to develop a HIV vaccine.

Modifying factors including: age, gender and level of education which would have been perceived to affect the volunteer’s decision to participate in a clinical trial have been shown in this study not to be significant factors.

This study found that volunteers were motivated by altruism as a primary motivator to help their family, society or world especially among volunteers who know of someone infected with HIV/AIDS. This was similar to a study in India which found that knowing a person with HIV infection acted as a motivator to volunteer in HIV vaccine trials [18]. However, a systematic review of 8 out of 12 studies reported that financial reward was the principal reason for participation in a HIV clinical trial [19]. Contrary findings were also made in studies in India and Brazil which observed that volunteers were of the opinion that monetary compensation was an entitlement to the participants which they would not forego as it was the principal motivator [20,21]. This difference in the principal motivator compared to our study could be because volunteers in our clinical trials only received re-imbursement as monetary compensation, which may not be enough to act as an incentive.

Personal benefits were cited as a secondary motivator to participation to get financial and health benefits. The desire to get health benefits could be linked to an individual’s perceived susceptibility of contracting HIV. Since the study was conducted in Nairobi, this desire could be of lower significance, as the city has a low prevalence of HIV as compared to western parts of Kenya. Contrary results were found in a study in China, where health benefits were the primary motivator for enrolling in a HIV vaccine trial [22]. The difference in this study could be attributed to the fact that the study participants in the China study were HIV positive hence the desire to delay enrolment to ART and reduce disease progression. Similar findings were made in Organization for Economic Co-operation and Development (OECD) countries [12] and Tanzania, where the desire to get protection from infection was secondary to collaboration with science as a few participants were expecting to get protection against HIV from the trial vaccine [23]

This study found that financial interest motivated younger volunteers while the need to help society and advance research motivated older volunteers; however there were no significant age differences among different motivators. This can be related to a study in South Africa which found that older and younger volunteers were equally motivated by altruistic reasons [24].

Most of the male and female volunteers were motivated by the desire to contribute to advancing research. Therefore, there was no evidence to link gender of volunteers and a specific motivator. It has been recognized that men and women tend to engage in different HIV risk behaviors hence there is a difference in their perceived susceptibility, and the resultant motivation to participate in HIV vaccine clinical trials, as well as their perception of benefits of the vaccine [25,26]. In India and Thailand, studies found that women were mostly motivated to participate in HIV vaccine trials to get protection against HIV infection via their husbands [27,28] which was not the finding of the present study.

The results indicated that the desire to advance research was the most common motivator across all education levels; however, there was no significant association between motivators and volunteer education level. Education level could be an indicator of the socio-economic status of the volunteers and the low financial rewards offered in trials might not make financial sense to the highly educated, assumed higher socio-economic group, leading to a disproportionate number of volunteers with lower education levels participating in clinical studies. Almeida et al. [29] found that volunteers with low education status were more likely to cite financial motivations to participate in a clinical study while Kass et al. [30], found contrary evidence that volunteers with higher education level were more likely to be motivated by financial benefits. This could be related to the type of financial incentives, such as whether it was reimbursement over costs incurred or payment in lieu of time spent in the trial.

## Limitations

1. The Health Belief Model is limited by the fact that behaviour is habitual and may inform the decision-making process and is sometimes performed for non-health related reasons such as social acceptability.
2. Use of a convenience sample diminished the generalizability of the study and exposed it to bias and high sampling error.
3. Social desirability bias as the questionnaire was administered by the nurse counsellors.
4. The study did not explore the relationship between the motivators and actual participation in HIV vaccine trials of the volunteers in the VPS and the relationship between motivators and retention in the clinical trials.

## Conclusion

The majority of volunteers in the HIV vaccine trials at our site were motivated by social benefits, suggesting that altruism can be a major contributor to participation in HIV vaccine studies. Personal benefit is a secondary motivator in participation in HIV vaccine clinical trials.

Volunteers willing to participate in future clinical trials as demonstrated by those in the VPS are mostly motivated by personal benefits while volunteers in the actual Phase I HIV vaccine clinical trials were mostly consistently motivated by social benefits. This may be explained as the volunteers in the VPS not assuming any immediate danger associated with vaccination, and thus enrol for their personal benefit. Those in the Phase I studies however, have perceived their susceptibility to HIV/AIDS, looked at the severity of the infection and benefits of participating in a HIV vaccine clinical trial and are cued to action.

The motivators to clinical trial participation were similar across age, education level and gender. Thus in a Sub-Saharan setting such as Nairobi, Kenya, targeting of volunteers for a HIV vaccine trial requires education on cues to action on what is required to prevent HIV/AIDS, (including volunteer participation), to develop an efficacious, accessible and affordable HIV vaccine.

## Supporting information

S1 Appendix Baseline demographics and reasons for volunteering form. (PDF)

## References

[1] UNAIDS. The Gap report. UNAIDS; 2015.

[2] National AIDS Control Council. Kenya AIDS Response Progress Report 2014: Progress towards Zero. [Internet]. 2014. Available: http://www.unaids.org/sites/default/files/country/documents/KEN_narrative_report_2014.pdf

[3] UNAIDS. UNAIDS report on the global AIDS epidemic, 2012. [Internet]. 2012. Available: http://www.unaids.org/sites/default/files/media_asset/20121120_UNAIDS_Global_Report_2012_with_annexes_en_1.pdf

[4] National AIDS and STI Control Programme. HIV/AIDS Decentralization Guidelines [Internet]. Nairobi, Kenya; 2009. Available: http://www.ilo.org/wcmsp5/groups/public/—ed_protect/—protrav/—ilo_aids/documents/legaldocument/wcms_127532.pdf

[5] National AIDS Control Council. Kenya AIDS Strategic Framework 2014/2015-2018/2019 [Internet]. 2014. Available: http://nacc.or.ke/wp-content/uploads/2015/09/KASF_Final.pdf

[6] Greco D, Diniz NM. Conflicts of interest in research involving human beings. J Int Bioethique Int J Bioeth. 2008;19: 143–154, 202–203. 10.3917/jib.191.0143 18664007

[7] Emanuel EJ, Wendler D, Killen J, Grady C. What Makes Clinical Research in Developing Countries Ethical? The Benchmarks of Ethical Research. J Infect Dis. 2004;189: 930–937. doi: 10.1086/381709 14976611 10.1086/381709

[8] Kochhar S. Challenges and impact of conducting vaccine trials in Asia and Africa,. Hum Vaccines Immunother. 2013;19: 924–927. 10.4161/hv.23405 PMC3903915 23321645

[9] Bhutta Z Ahmed. Ethics in international health research: a perspective from the developing world,. Bull World Health Organ. 2002;80: 114–120. 11953789 PMC2567726

[10] Andanda P, Lucas J Cook. Majengo HIV/AIDS Research Case: A Report for GenBenefit (2007) [Internet]. 2007. Available: https://www.uclan.ac.uk/research/explore/projects/assets/cpe_genbenefit_nairobi_case.pdf

[11] Isler MR, Miles MS, Banks B, Corbie-Smith G. Acceptability of a Mobile Health Unit for Rural HIV Clinical Trial Enrollment and Participation. AIDS Behav. 2012;16: 1895–1901. doi: 10.1007/s10461-012-0151-z 22350829 10.1007/s10461-012-0151-z PMC3763695

[12] Dhalla S, Poole G. Motivators to Participation in Actual HIV Vaccine Trials. AIDS Behav. 2014;18: 263–277. doi: 10.1007/s10461-013-0519-8 23736885 10.1007/s10461-013-0519-8

[13] Center for Disease Control, “Center for Disease Control and Prevention Kenya Annual Report,.” 2015.

[14] African Population and Health Research Center (APHRC). Population and Health Dynamics in Nairobi’s Informal Settlements: Report of the Nairobi Cross-sectional Slums Survey (NCSS) 2012, [Internet]. Center, Nairobi, Kenya: African Population and Health Research Center; 2014 Available: http://aphrc.org/wp-content/uploads/2014/08/NCSS2-FINAL-Report.pdf

[15] Krueger RA, Casey MA. Focus groups: a practical guide for applied research. 5th edition Thousand Oaks, California: SAGE; 2015.

[16] Ryan GW, Bernard HR. Techniques to Identify Themes. Field Methods. 2003;15: 85–109. doi: 10.1177/1525822X02239569

[17] Rimer B, Glanz K. Theory at a glance: A guide for health promotion practice. [Internet]. National Cancer Institute; 1997 Available: http://www.sbccimplementationkits.org/demandrmnch/wp-content/uploads/2014/02/Theory-at-a-Glance-A-Guide-For-Health-Promotion-Practice.pdf

[18] Sahay S, Kumar M, Srikrishnan AK, Ramanathan V, Mehendale S. Experiences in recruiting volunteers through community based initiatives in Phase-1 vaccine trials in India. Hum Vaccines Immunother. 2014;10: 485–491. doi: 10.4161/hv.26799 24141176 10.4161/hv.26799 PMC4185896

[19] Stunkel L, Grady C. More than the money: A review of the literature examining healthy volunteer motivations. Contemp Clin Trials. 2011;32: 342–352. doi: 10.1016/j.cct.2010.12.003 21146635 10.1016/j.cct.2010.12.003 PMC4943215

[20] Chakrapani V, Newman PA, Singhal N, Jerajani J, Shunmugam M. Willingness to Participate in HIV Vaccine Trials among Men Who Have Sex with Men in Chennai and Mumbai, India: A Social Ecological Approach. Cameron DW, editor. PLoS ONE. 2012;7: e51080 doi: 10.1371/journal.pone.0051080 23226560 10.1371/journal.pone.0051080 PMC3514227

[21] Nappo SA, Iafrate GB, Sanchez ZM. Motives for participating in a clinical research trial: a pilot study in Brazil. BMC Public Health. 2013;13 doi: 10.1186/1471-2458-13-19 23302375 10.1186/1471-2458-13-19 PMC3554489

[22] Dong Y, Shen X, Guo R, Liu B, Zhu L, Wang J, et al Willingness to Participate in HIV Therapeutic Vaccine Trials among HIV-Infected Patients on ART in China. Jin X, editor. PLoS ONE. 2014;9: e111321 doi: 10.1371/journal.pone.0111321 25372044 10.1371/journal.pone.0111321 PMC4221013

[23] Tarimo EAM, Bakari M, Kakoko DCV, Kohi TW, Mhalu F, Sandstrom E, et al Motivations to participate in a Phase I/II HIV vaccine trial: A descriptive study from Dar es Salaam, Tanzania. BMC Public Health. 2016;16 doi: 10.1186/s12889-016-2875-6 26911203 10.1186/s12889-016-2875-6 PMC4765221

[24] Volk JE, Hessol NA, Gray GE, Kublin JG, Churchyard GJ, Mlisana K, et al The HVTN503/Phambili HIV vaccine trial: a comparison of younger and older participants. Int J STD AIDS. 2014;25: 332–340. doi: 10.1177/0956462413506892 24104693 10.1177/0956462413506892 PMC3968181

[25] Mehrotra P, Noar SM, Zimmerman RS, Palmgreen P. Demographic and Personality Factors as Predictors of HIV/STD Partner-Specific Risk Perceptions: Implications for Interventions. AIDS Educ Prev. 2009;21: 39–54. doi: 10.1521/aeap.2009.21.1.39 19243230 10.1521/aeap.2009.21.1.39 PMC4546104

[26] Bass SB, Wolak C, Greener J, Tedaldi E, Nanavati A, Ruppert K, et al Using perceptual mapping methods to understand gender differences in perceived barriers and benefits of clinical research participation in urban minority HIV+ patients. AIDS Care. 2016;28: 528–536. doi: 10.1080/09540121.2015.1112352 26572215 10.1080/09540121.2015.1112352

[27] Suhadev M, Nyamathi AM, Swaminathan S, Venkatesan P, Raja Sakthivel M, Shenbagavalli R, et al A pilot study on willingness to participate in future preventive HIV vaccine trials. Indian J Med Res. 2006;124: 631–640. 17287550

[28] Liamputtong P, Haritavorn N, Kiatying-Angsulee N. Participating in HIV Clinical Trials: Reasons and Experiences Among Women Living With HIV in Thailand. J HIVAIDS Soc Serv. 2015;14: 239–256. doi: 10.1080/15381501.2014.912175

[29] Almeida L, Azevedo B, Nunes T, Vaz-da-Silva M, Soares-da-Silva P. Why healthy subjects volunteer for phase I studies and how they perceive their participation? Eur J Clin Pharmacol. 2007;63: 1085–1094. doi: 10.1007/s00228-007-0368-3 17891536 10.1007/s00228-007-0368-3

[30] Kass NE, Myers R, Fuchs EJ, Carson KA, Flexner C. Balancing Justice and Autonomy in Clinical Research With Healthy Volunteers. Clin Pharmacol Ther. 2007;82: 219–227. doi: 10.1038/sj.clpt.6100192 17410122 10.1038/sj.clpt.6100192

