# Supplementary material for: Volunteer motivators for participating in HIV vaccine clinical trials in Nairobi, Kenya
Source: PLoS One. 2017 Sep 7;12(9):e0183788. doi: 10.1371/journal.pone.0183788 (PMC5589082; doi:10.1371/journal.pone.0183788)
Supplement: S1 Appendix — (PDF) [file pone.0183788.s001.pdf]

BASELINE DEMOGRAPHICS & REASONS FOR VOLUNTEERING FORM

(SCREENING VISIT)

Volunteer ID No.: ___________________________

Visit Date: ¦___¦___¦/¦___¦___¦___¦/¦___¦___¦___¦___¦ (DD / MMM / YYYY)

Gender: Male ☐ Female ☐

1. What is the subject's date of birth? (dd/mmm/yyyy) ¦__¦__¦ / ¦__¦__¦__¦ / ¦__¦__¦__¦__¦
2. What is the subject's age?

   Fixed Unit: years ___________________________

3. Marital status:
   - ☐ Single- Never married.
   - ☐ Married
   - ☐ Separated/Divorced
   - ☐ Widowed
   - ☐ Other; please specify ______________________________________
4. Apart from your housework how would you describe your current work situation?
   - ☐ Unemployed
   - ☐ Salaried job
   - ☐ Self-employed professional
   - ☐ Casual worker
   - ☐ Other; please specify ____
5. Have you ever attended school? [ ] Yes [ ] No If yes, how many total years of school have you completed in each category? (Enter "00" if no education in that category.)

   Primary School [ ][ ]
   Secondary School [ ][ ]
   Tertiary Education [ ][ ] Specify: _______________________________________

6. Have you disclosed your participation? [ ] Yes [ ] No
   If Yes who have you shared the information with?
   - ☐ Spouse
   - ☐ Friend
   - ☐ Family member (specify) _____________
   - ☐ Other (specify) _____________

7. What was their reaction?
____
____

8. What are your reasons for volunteering?
____
____

9. What do you expect to benefit from participating in this study?
____
____

Form Completed by	____________________ Signature	____________________ Printed Name/Initials	____________________ Date Form Signed
Reviewed by	____________________ Signature	____________________ Printed Name/Initials	____________________ Date Form Signed
